# IL10 inhibits starvation-induced autophagy in hypertrophic scar fibroblasts via cross talk between the IL10-IL10R-STAT3 and IL10-AKT-mTOR pathways

**DOI:** 10.1038/cddis.2016.44

**Published:** 2016-03-10

**Authors:** J Shi, H Wang, H Guan, S Shi, Y Li, X Wu, N Li, C Yang, X Bai, W Cai, F Yang, X Wang, L Su, Z Zheng, D Hu

**Affiliations:** 1 Department of Burns and Cutaneous Surgery, Xijing Hospital, Fourth Military Medical University, 127 West Chang-le Road, Xi'an 710032, China

## Abstract

Hypertrophic scar (HS) is a serious skin fibrotic disease characterized by excessive hypercellularity and extracellular matrix (ECM) component deposition. Autophagy is a tightly regulated physiological process essential for cellular maintenance, differentiation, development, and homeostasis. Previous studies show that IL10 has potential therapeutic benefits in terms of preventing and reducing HS formation. However, no studies have examined IL10-mediated autophagy during the pathological process of HS formation. Here, we examined the effect of IL10 on starvation-induced autophagy and investigated the molecular mechanism underlying IL10-mediated inhibition of autophagy in HS-derived fibroblasts (HSFs) under starvation conditions. Immunostaining and PCR analysis revealed that a specific component of the IL10 receptor, IL10 alpha-chain (IL10R*α*), is expressed in HSFs. Transmission electron microscopy and western blot analysis revealed that IL10 inhibited starvation-induced autophagy and induced the expression of p-AKT and p-STAT3 in HSFs in a dose-dependent manner. Blocking IL10R, p-AKT, p-mTOR, and p-STAT3 using specific inhibitors (IL10RB, LY294002, rapamycin, and cryptotanshinone, respectively) showed that IL10 inhibited autophagy via IL10R*α*-mediated activation of STAT3 (the IL10R-STAT3 pathway) and by directly activating the AKT-mTOR pathway. Notably, these results suggest that IL10-mediated inhibition of autophagy is facilitated by the cross talk between STAT3, AKT, and mTOR; in other words, the IL10-IL10R-STAT3 and IL10-AKT-mTOR pathways. Finally, the results also indicate that mTOR-p70S6K is the molecule upon which these two pathways converge to induce IL10-mediated inhibition of autophagy in starved HSFs. In summary, the findings reported herein shed light on the molecular mechanism underlying IL10-mediated inhibition of autophagy and suggest that IL10 is a potential therapeutic agent for the treatment of HS.

Autophagy is a degradative process in eukaryotic cells that removes or turns over bulk cytoplasmic constituents through the endosomal and lysosomal fusion system (i.e., autophagosomes).^1, 2^ Autophagy is induced by stressful conditions such as starvation and pathogenic invasion.^2^

Hypertrophic scar (HS) is a major skin fibrotic disorder caused by hypercellularity and extracellular matrix (ECM) component deposition.^3, 4, 5^ HS formation is usually recognized as the consequence of disturbed tissue repair processes and/or disrupted homeostasis in the skin after traumatic injury: HS negatively impacts on patient appearance, skeletal muscle function, and quality of life in general.^6, 7, 8, 9^ About 40–70% of surgeries and over 91% of burn injuries result in HS.^10^ A key feature of HS is a metabolic disorder of collagen-based ECM proteins.^11, 12, 13^ Autophagy has an important role in homeostasis of tissue structure and function.^2, 14, 15^ Skin autophagic capability is associated with HS and with the pathogenesis of many human diseases.^16, 17, 18, 19, 20, 21, 22, 23^

Existing studies suggest that cytokines are important regulators of the autophagic process in both immune and non-immune cells.^24, 25, 26^ Interleukin-10 (IL10), expressed by a variety of mammalian cell types, was first described as a cytokine-synthesis-inhibitory factor with immunosuppressive and anti-inflammatory functions.^27, 28^ IL10 has a pivotal role in wound healing^29, 30^ and is a promising therapeutic agent for scar improvement in both animal models and human cutaneous wounds.^9, 31, 32^

Fibroblasts are one of the most important effector cells responsible for HS formation.^12, 33, 34^ Thus, we were prompted to elucidate the mechanisms underlying the interactions among IL10, autophagy, and HS formation, with the aim of providing a molecular foundation for the therapeutic efficacy IL10. We used HS tissue, HS-derived fibroblasts (HSFs), and starvation-induced autophagy in HSFs as our research platform.

Here, we report that IL10 inhibited autophagy by interfering with IL10R-mediated activation of IL10R-STAT3, as well as by activating the AKT-mTOR pathway. In addition, cross talk among STAT3, AKT, and mTOR and between the IL10-IL10R-STAT3 and IL10-AKT-mTOR pathways collaboratively regulated starvation-induced autophagy in HSFs.

## Results

### IL10 inhibits starvation-induced autophagy in HSFs

**Figure 1:**
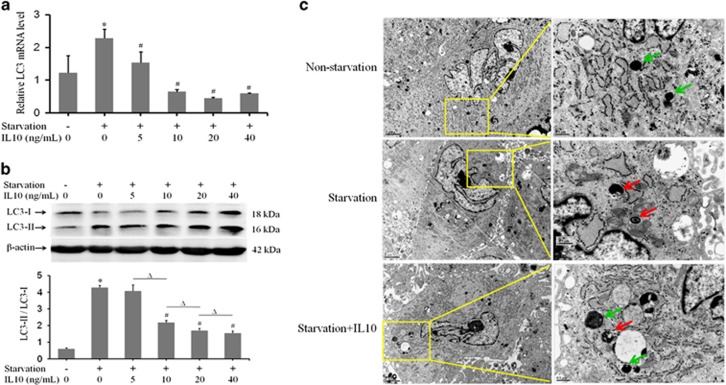
IL10-mediated inhibition of starvation-induced autophagy in HSFs. HSFs (70–80% confluent) were starved by culturing in serum-depleted medium for 12–16 h prior to exposure to different doses of IL10 for 6 h. (**a**) The amount of LC3 mRNA was analyzed by qRT-PCR (data are expressed as the mean±S.E.M.; *n*=3; *P<0.05 *versus* the non-starvation group; ^#^*P*<0.05 *versus* the starvation group). (**b**) LC3-I and LC3-II protein expression was analyzed by western blotting and the LC3-II/LC3-I ratio calculated based on signal intensity (*n*=3; *P<0.05 *versus* the non-starvation group; ^#^*P*<0.05 *versus* the starvation group; Δ*P*<0.05). (**c**) Few autophagosomes were observed in the non-starvation group (upper row, green arrows indicate lysosomes). Autophagosome numbers in the starved group (middle row, red arrows) increased, whereas they decreased in cells treated with 20 ng/ml IL10 (lower row). Scale bars, 2 and 0.5 *μ*m

To test whether IL10 affects autophagy in HSFs, we subjected the cells to serum starvation in the absence or presence of IL10. Expression of the autophagy marker, LC3, in HSFs was then examined by qRT-PCR. As shown in Figure 1a, LC3 expression was upregulated by about twofold in starved HSFs (*P*=0.00014; Figure 1a) in comparison with non-starved controls. Interestingly, LC3 expression in starved HSFs was downregulated by IL10 at concentrations as low as 5 ng/ml (*P*=0.00096); such downregulation was more pronounced at higher concentrations (10, 20, and 40 ng/ml; *P*=0.00001, *P*=0.00001, and *P*=0.00001, respectively; Figure 1a). It is noteworthy that IL10's inhibitory effect on LC3 expression reached a plateau at 10 ng/ml. Western blot analysis of LC3 isoforms (LC3-I and LC3-II) indicated that the LC3-II/LC3-I ratio was markedly higher in starved HSFs than in non-starved controls (*P*=0.00000; Figure 1b). Intriguingly, the LC3-II/LC3-I ratio was similar to Figure 1a in the presence of IL10 at 10 ng/ml (*P*=0.18450), but was downregulated by higher concentrations of IL10 (*P*=0.00005, *P*=0.00003, and *P*=0.00001, respectively; Figure 1b). Likewise, the LC3-II/LC3-I ratio was comparable at the three higher concentrations of IL10. These results show that IL10 inhibits expression of the autophagy molecular marker LC3 in starved HSFs.

To verify the effect of IL10 on autophagy in starved HSFs, we examined autophagosome formation using transmission electron microscopy. Ultra-structure analysis showed that the number of autophagosomes in starved HSFs was substantially higher (Figure 1c, middle row, red arrows) than that in non-starved controls (Figure 1c, upper row). Although the number of autophagosomes in starved HSFs treated with 20 ng/ml IL10 (Figure 1c, lower row; green arrow indicates a lysosome) was much lower than that in starved HSFs, it was still higher than that in the control. These results indicate that starvation induces the formation of autophagosomes in HSFs, and the process is inhibited by IL10.

### IL10R*α* expression in HS tissues and HSFs

**Figure 2:**
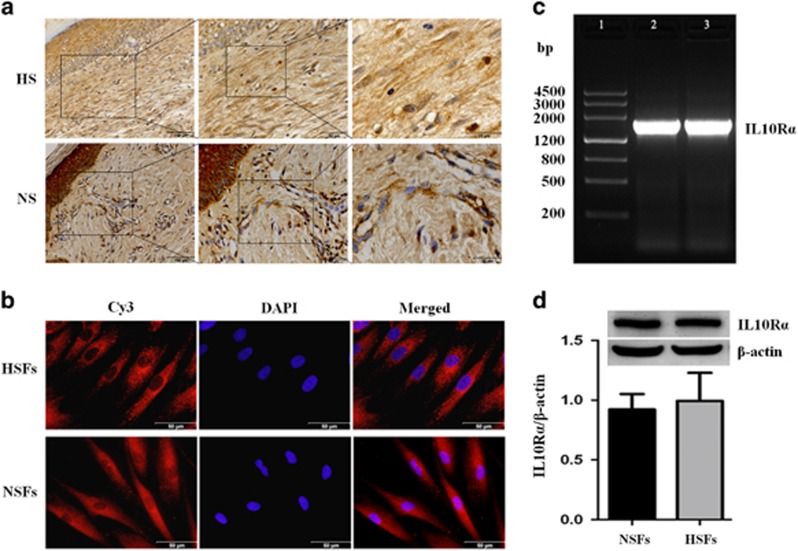
IL10R*α* expression in HS tissues and HSFs. (**a**) IL10R*α* expression was detected by streptavidin-peroxidase DAB staining of HS and NS tissues. Scale bars=100, 50, and 25 *μ*m. (**b**) The cellular localization of IL10R*α* was analyzed using a specific mAb and a Cy3-conjugated secondary antibody. Fibroblast nuclei were stained with DAPI. Scale bars, 50 *μ*m. (**c**) PCR was performed to analyze IL10R*α* mRNA expression in HSFs and NSFs. Lane 1, DNA ladder; lane 2, IL10R*α* in HSFs; lane 3, IL10R*α* in NSFs. (**d**) IL10R*α* protein (molecular weight, 63 kDa) expression in HSFs (hatched bar) and NSFs (closed bar). Data are expressed as the mean±S.E.M. (*n*=3; *P*>0.05 *versus* the NSFs group)

Immunostaining of IL10R *α*-chain (IL10R*α*) showed that, in HS and HSFs, IL10R*α* was localized both on the cell surface and in the cytoplasm (Figures 2a and b), a distribution and localization similar to that in normal skin (NS) and normal skin-derived fibroblasts (NSFs; Figures 2a and b).

PCR analysis of the IL10R*α* gene amplified a fragment of 1736 bp from both HSFs and NSFs (Figure 2c). Sequence analysis verified that both amplified fragments were derived from human IL10R*α* (NM-001558.3, data not shown). As expected, western blotting detected the expression of a 63 kDa IL10R*α* protein at similar levels in both HSFs and NSFs (*P*=0.25480; Figure 2d), as well as in HS and NS (data not shown).

### IL10-mediates activation of AKT and STAT3 in starved HSFs

**Figure 3:**
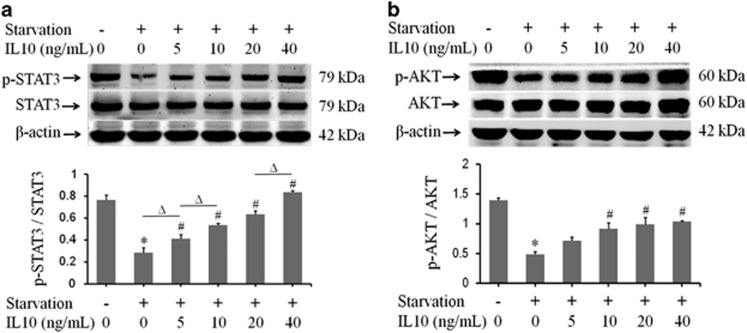
IL10-mediated activation of STAT3 and AKT in starved HSFs. HSFs (70–80% confluent) were starved by culturing in serum-depleted medium for 12–16 h before exposure to different concentrations of IL10 for 30 min. (**a**) p-STAT3 and STAT3 protein expression and changes in the p-STAT3/STAT3 ratio (data expressed as the mean±S.E.M.; *n*=3, *P<0.05 *versus* the non-starvation control and ^#^*P*<0.05 *versus* the starvation control; Δ*P*<0.05). (**b**) p-AKT and AKT protein expression and changes in the p-AKT/AKT ratio (*n*=3; *P<0.05 *versus* the non-starvation control and ^#^*P*<0.05 *versus* the starvation control)

IL10 activates the IL10R-STAT3 signaling pathway by binding to IL10 receptors (IL10R) on the cell surface.^11, 35, 36^ To determine whether IL10 inhibits autophagy in HSFs via the IL10R-STAT3 signaling pathway, the expression of p-STAT3 and STAT3 was analyzed in starved HSFs in the absence or presence of different concentrations of IL10 (Figure 3). Western blot analysis revealed that starvation downregulated p-STAT3 expression (*P*=0.00020; Figure 3a), and that IL10 alleviated the inhibitory effect of starvation on p-STAT3. By contrast, STAT3 expression appeared unaffected by either starvation or IL10 (Figure 3a). Interestingly, the antagonistic role of IL10 on starvation-mediated inhibition of p-STAT3 expression was dose-dependent (*P*=0.02531, *P*=0.001392, *P*=0.00114, and *P*=0.00004; Figure 3a), and dose-dependently increased (Δ*P*<0.05; Figure 3a).

The AKT-mTOR pathway is essential for fundamental cellular function.^37, 38, 39^ Next, we examined whether the AKT-mTOR pathway is involved in IL10-mediated inhibition of autophagy in HSFs. Western blot analysis of AKT and p-AKT expression revealed that starvation and IL10 had similar effects (*P*=0.00002, *P*=0.06549, *P*=0.01574, *P*=0.01237, and *P*=0.00006; Figure 3b). Taken together, these results indicate that IL10 inhibits autophagy via both the IL10R-STAT3 and AKT-mTOR pathways.

### IL10 inhibits autophagy via the IL10/IL10R-STAT3 pathway

**Figure 4:**
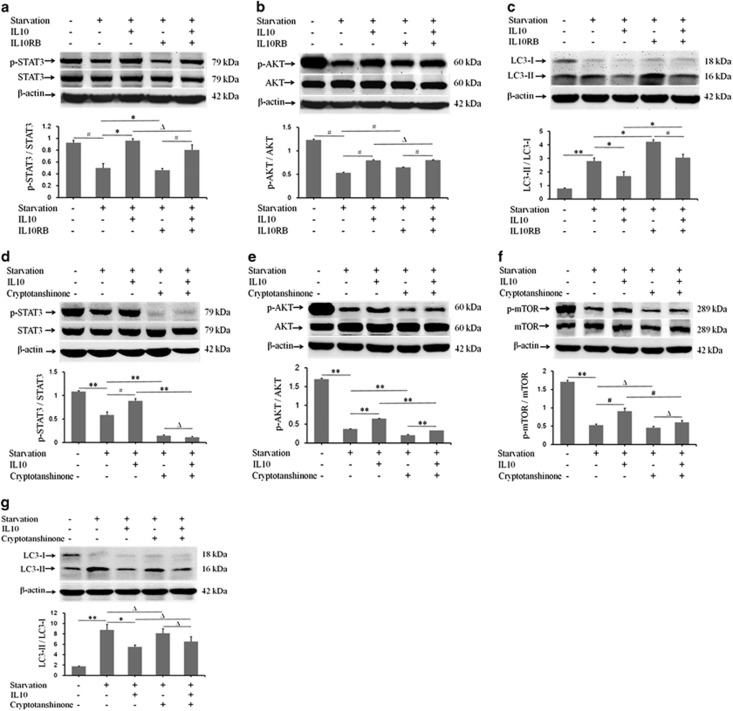
IL10-mediated inhibition of autophagy via the IL10/IL10R-STAT3 pathway. HSFs (70–80% confluent) were starved by culturing in serum-depleted medium for 12–16 h. p-STAT3 and p-AKT protein expression in response to IL10 treatment was then assessed after incubation with IL10RB for 30 min. (**a**) p-STAT3 and STAT3 protein expression and changes in the p-STAT3/STAT3 ratio in the presence of IL10 and/or IL10RB (data are expressed as the mean±S.E.M.; *n*=3; *P<0.05 *versus* the starvation control and ^#^*P*<0.01 *versus* the IL10RB treatment group). (**b**) p-AKT and AKT protein expression and changes in the p-AKT/AKT ratio in the presence of IL10 and/or IL10RB (*n*=3; ^#^*P*<0.01 *versus* the starvation control and ^#^*P*<0.01 *versus* the IL10RB treatment group). (**c**) LC3-I and LC3-II protein expression and changes in the LC3-II/LC3-I ratio in the presence of IL10 and/or IL10RB (*n*=3; *P<0.05 *versus* the starvation control; *P<0.05 *versus* the starvation group; ^#^*P*<0.01 *versus* the IL10RB treatment group). (**d**) p-STAT3 and STAT3 protein expression and changes in the p-STAT3/STAT3 ratio in the presence of IL10 and/or the STAT3 inhibitor, cryptotanshinone (*n*=3; **P<0.001 *versus* the starvation control; ^#^*P*<0.01 *versus* the starvation group; Δ*P*>0.05 *versus* the cryptotanshinone-treatment group). (**e**) p-AKT and AKT protein expression and changes in the p-AKT/AKT ratio in the presence of IL10 and/or cryptotanshinone (*n*=3; **P<0.001 *versus* the starvation control or the cryptotanshinone-treated group). (**f**) p-mTOR and mTOR protein expression and changes in the p-mTOR/mTOR ratio the presence of IL10 and/or cryptotanshinone (*n*=3; Δ*P*>0.05 *versus* the starvation control or cryptotanshinone-treated groups and ^#^*P*<0.01 *versus* the starvation control group). (**g**) LC3-I and LC3-II protein expression and changes in the LC3-II/LC3-I ratio in the presence of IL10 and/or cryptotanshinone (*n*=3; Δ*P*>0.05 *versus* the starvation control or cryptotanshinone-treated groups and *P<0.05 *versus* the starvation control group)

To verify involvement of the IL10R-STAT3 pathway in IL10-mediated inhibition of autophagy in starved HSFs, the expression of p-STAT3/STAT3, p-AKT/AKT, and LC3-II/LC3-I in starved HSFs was compared in the presence/absence of IL10 and in the presence/absence of an anti-IL10R antibody (IL10RB). As shown in Figure 4, IL10RB reversed IL10-mediated effects on the expression of p-STAT3 (*P*=0.04779, Figure 4a) and p-AKT (*P*=0.00011, Figure 4b) and on the ratio of LC3-II/LC3-I (*P*=0.00262, Figure 4c) in starved HSFs. These results indicate that IL10-mediated inhibition of autophagy in starved HSFs is at least partially IL10R*α*-dependent.

To further understand the signal transduction pathway via which IL10 inhibits autophagy in starved HSFs, cryptotanshinone (a specific p-STAT3 inhibitor) was applied to starved HSFs in the presence/absence of IL10. Western blot analysis of p-STAT3/STAT3, p-AKT/AKT, LC3-II/LC3-I, and p-mTOR/mTOR showed that cryptotanshinone substantially suppressed IL10-induced p-STAT3 expression (*P*=0.00022, Figure 4d), and (albeit to a lesser extent) that of IL10-induced p-AKT expression (*P*=0.00026, Figure 4e), despite the presence of IL10; however, p-mTOR expression was unchanged (*P*=0.13835; Figure 4f). Nonetheless, cryptotanshinone did not significantly alter the LC3-II/LC3-I ratio, which was reduced in the presence of IL10 (*P*=0.58288; Figure 4g). These results suggest that IL10-mediated inhibition of autophagy in starved HSFs involves p-STAT3/STAT3, p-AKT/AKT, and p-mTOR/mTOR, probably via the IL10-IL10R-STAT3 and IL10-AKT-mTOR pathways.

### IL10 inhibits autophagy via the IL10-AKT-mTOR pathway

**Figure 5:**
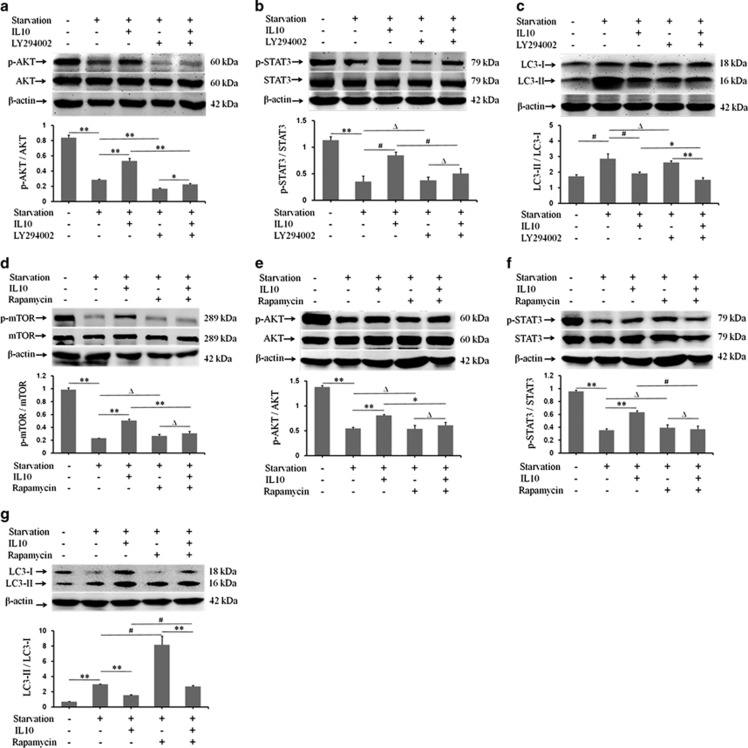
IL10-mediated inhibition of autophagy via the IL10/AKT-mTOR pathway. HSFs (70–80% confluent) were starved by culturing in serum-depleted medium for 12–16 h. p-AKT, p-STAT3, and p-mTOR protein expression in response to IL10 was assessed after incubation with LY294002 or rapamycin for 30 min. (**a**) p-AKT and AKT protein expression and changes in the p-AKT/AKT ratio in the presence of IL10 and/or the p-AKT inhibitor LY294002 (data are expressed as the mean±S.E.M.; *n*=3; **P<0.001 *versus* the starvation control and *P<0.05 *versus* the LY294002 treatment group). (**b**) p-STAT3 and STAT3 protein expression and changes in the p-STAT3/STAT3 ratio in the presence of IL10 and/or LY294002 (*n*=3; Δ*P*>0.05 *versus* the starvation and LY294002 treatment groups). (**c**) LC3-I and LC3-II protein expression and changes in the LC3-II/LC3-I ratio in the presence of IL10 and/or Ly294002 (*n*=3; Δ*P*>0.05 *versus* the starvation group; **P<0.001 *versus* the LY294002 treatment group). (**d**) p-mTOR and mTOR protein expression and changes in the p-mTOR/mTOR ratio in the presence of IL10 and/or the p-mTOR inhibitor, rapamycin (data are expressed as the mean±S.E.M.; *n*=3; Δ*P*>0.05 *versus* the starvation and rapamycin-treatment groups). (**e**) p-AKT and AKT protein expression and changes in the p-AKT/AKT ratio in the presence of IL10 and/or rapamycin (*n*=3; Δ*P*>0.05 *versus* the starvation and rapamycin-treatment groups). (**f**) p-STAT3 and STAT3 protein expression and changes in the p-STAT3/STAT3 ratio in the presence of IL10 and/or rapamycin (*n*=3; Δ*P*>0.05 *versus* the starvation and rapamycin-treatment groups). (**g**) LC3-I and LC3-II protein expression and changes in the LC3-II/LC3-I ratio in the presence of IL10 and/or rapamycin (*n*=3; ^#^*P*<0.01 *versus* the starvation group and **P<0.001 *versus* the rapamycin-treatment group)

To examine the role of the IL10-AKT-mTOR pathway in IL10-mediated inhibition of autophagy in starved HSFs, LY294002 (a specific inhibitor of p-AKT) was applied to starved HSFs in the presence/absence of IL10. Western blot analysis of p-AKT/AKT, p-STAT3/STAT3, and LC3-II/LC3-I ratios showed that LY294002 suppressed IL10-induced p-AKT expression (*P*=0.00007; Figure 5a) and, to a lesser extent, downregulated IL10-induced p-STAT3 expression (*P*=0.00715; Figure 5b); it also covered IL10-mediated reduction in the LC3-II/LC3-I ratio (*P*=0.01716; Figure 5c).

To examine the involvement of p-mTOR in IL10-mediated inhibition of autophagy in starved HSFs, rapamycin (a specific mTOR inhibitor) was applied to starved HSFs in the presence or absence of IL10. Western blot analysis of p-mTOR/mTOR, p-AKT/AKT, p-STAT3/STAT3, and LC3-II/LC3-I ratios showed that rapamycin suppressed IL10-mediated upregulation of p-mTOR (*P*=0.00034; Figure 5d), p-AKT (*P*=0.04379; Figure 5e), and p-STAT3 (*P*=0.00444; Figure 5f) expression. However, rapamycin significantly increased the LC3-II/LC3-I ratio (*P*=0.00228; Figure 5g). These results imply that p-mTOR is a key and signaling molecule involved in IL10-mediated inhibition of autophagy in starved HSFs because inhibiting p-mTOR affects both the AKT-mTOR and IL10R-STAT3 pathways.

### IL10 inhibits autophagy via cross talk between the AKT-mTOR and IL10R-STAT3 pathways

**Figure 6:**
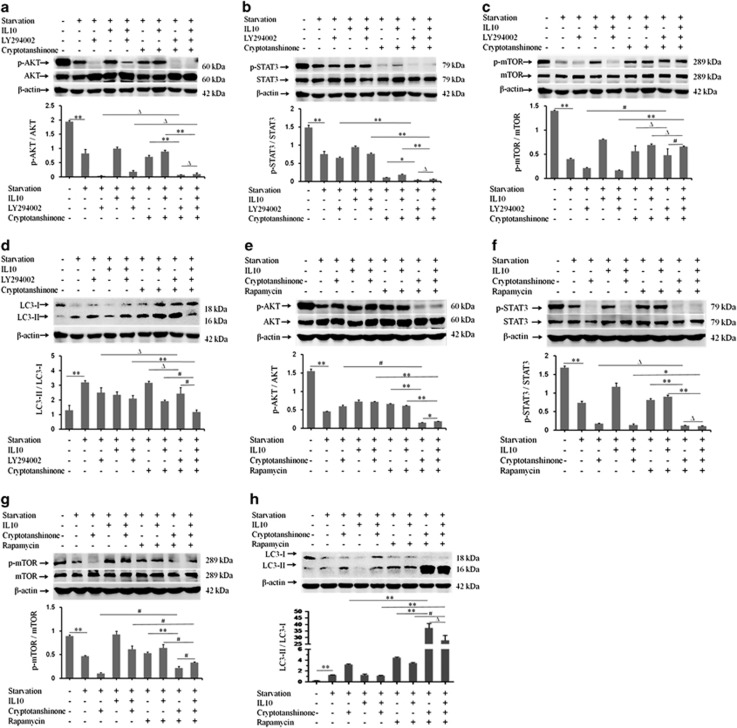
IL10-mediated inhibition of autophagy via cross talk between the AKT-mTOR and IL10R-STAT3 pathways. HSFs (70–80% confluent) were starved by culturing in serum-depleted medium for 12–16 h. p-AKT, p-STAT3, and p-mTOR protein expression in response to IL10 was then assessed after treatment with LY294002, cryptotanshinone, and rapamycin (or combinations thereof) for 30 min. (**a**) p-AKT and AKT protein expression and changes in the p-AKT/AKT ratio in the presence of IL10, LY294002, or cryptotanshinone (or combinations thereof) (data are expressed as the mean±S.E.M.; *n*=3; Δ*P*>0.05 and **P<0.001 *versus* individual treatment groups; Δ*P*>0.05 and **P<0.001 *versus* the LY294002+cryptotanshinone or cryptotanshinone-treatment groups, and Δ*P*>0.05 *versus* the LY294002 treatment group). (**b**) p-STAT3 and STAT3 protein expression and changes in the p-STAT3/STAT3 ratio in the presence of IL10, LY294002, or cryptotanshinone (or combinations thereof) (*n*=3; **P<0.001 *versus* individual treatment groups and Δ*P*>0.05 and **P<0.001 *versus* the LY294002+cryptotanshinone or individual treatment groups). (**c**) p-mTOR and mTOR protein expression and changes in the p-mTOR/mTOR ratio in the presence of IL10, LY294002, or cryptotanshinone (or combinations thereof) (*n*=3; ^#^*P*<0.01 *versus* the LY294002-treated or LY294002+cryptotanshinone-treated groups; Δ*P*>0.05 *versus* the cryptotanshinone-treated group and **P<0.001 *versus* the LY294002-treated group). (**d**) LC3-I and LC3-II protein expression and changes in the LC3-II/LC3-I ratio in the presence of IL10, LY294002, or cryptotanshinone (or combinations thereof) (*n*=3; Δ*P*>0.05 *versus* the cryptotanshinone- or LY294002-treated groups; **P<0.001 *versus* the group LY294002-treated group; and ^#^*P*<0.01 *versus* the LY294002+cryptotanshinone-treated group). (**e**) p-AKT and AKT protein expression and changes in the p-AKT/AKT ratio in the presence of IL10, cryptotanshinone, or rapamycin (or combinations thereof) (*n*=3; **P<0.001 and ^#^*P*<0.01 *versus* the individual treatment groups; *P<0.05 *versus* the cryptotanshinone+rapamycin-treated group). (**f**) p-STAT3 and STAT3 protein expression and changes in the p-STAT3/STAT3 ratio in the presence of IL10, cryptotanshinone, or rapamycin (or combinations thereof) (*n*=3; **P<0.001 *versus* the rapamycin-treated group; Δ*P*>0.05 *versus* the cryptotanshinone+rapamycin-treated group). (**g**) p-mTOR and mTOR protein expression and changes in the p-mTOR/mTOR ratio in the presence of IL10, cryptotanshinone, or rapamycin (or combinations thereof) (*n*=3; **P<0.001 and ^#^*P*<0.01 *versus* the individual treatment groups or the cryptotanshinone+rapamycin-treated group). (**h**) LC3-I and LC3-II protein expression and changes in the LC3-II/LC3-I ratio in the presence of IL10, cryptotanshinone, or rapamycin (or combinations thereof) (*n*=3; **P<0.001 *versus* the starvation control and individual treatment groups; ^#^*P*<0.01 *versus* the cryptotanshinone+rapamycin-treated or individual treatment groups; and Δ*P*>0.05 *versus* the cryptotanshinone+rapamycin-treated group)

The involvement of both the AKT-mTOR and IL10R-STAT3 pathways in IL10-mediated inhibition of starvation-induced autophagy in HSFs led us to hypothesize that these two pathways interact to facilitate IL10-mediated inhibition of autophagy. To test this, starved HSFs were treated with LY294002/rapamycin or with cryptotanshinone/rapamycin in the presence/absence of IL10. Western blot analysis of p-AKT/AKT, p-STAT3/STAT3, p-mTOR/mTOR, and LC3-II/LC3-I showed that, as expected, LY294002/cryptotanshinone almost completely abolished p-AKT (*P*=0.11713 and *P*=0.00000; Figure 6a) and p-STAT3 (*P*=0.00000 and *P*=0.00002; Figure 6b), even if in the presence of IL10. Whereas LY294002 alone significantly suppressed IL10-mediated upregulation of p-AKT expression, cryptotanshinone alone failed to suppress starvation-induced or IL10-mediated p-AKT expression in starved HSFs independent of IL10 (Figure 6a). By contrast, LY294002 alone had no significant effect on p-STAT3 expression in the presence or absence of IL10, whereas cryptotanshinone strongly suppressed p-STAT3 expression in starved HSFs independently of IL10 (Figure 6b). More interestingly, LY294002 alone downregulated p-mTOR expression in a manner similar to p-AKT (Figures 6a and c), whereas cryptotanshinone upregulated starvation-induced p-mTOR expression in the absence of IL10 (*P*=0.00509; Figure 6c). In the absence of IL10, both LY294002 and cryptotanshinone (either alone or together) failed to increase the LC3-II/LC3-I ratio (*P*=0.12734; Figure 6d), whereas in the presence of IL10, cryptotanshinone (either alone or together with LY294002) further reduced the LC3-II/LC3-I ratio (*P*=0.00840; Figure 6d).

The combination of cryptotanshinone and rapamycin strongly suppressed expression of p-AKT (*P*=0.00006 and *P*=0.00004; Figure 6e), p-STAT3 (*P*=0.00009 and *P*=0.33470; Figure 6f), and p-mTOR (*P*=0.00045 and *P*=0.00125; Figure 6g), and abolished LC3-I expression while at the same time markedly increasing LC3-II expression, resulting in a striking increase in the LC3-II/LC3-I ratio (*P*=0.00074 and *P*=0.00064; Figure 6h) in starved HSFs. Similar to the observations described in Figure 6a, cryptotanshinone alone alleviated starvation-induced suppression of p-AKT expression in the presence or absence of IL10 (Figure 6e), as did rapamycin (Figure 6e).

Furthermore, consistent with the observations in Figure 6b, cryptotanshinone alone strongly inhibited p-STAT3 expression, even in the presence of IL10 (*P*=0.35483; Figure 6f). By contrast, rapamycin alone slightly reversed starvation-induced suppression of p-STAT3 expression (Figure 6f). Cryptotanshinone, either alone or together with rapamycin, either abolished or strongly inhibited p-mTOR expression in the absence of IL10 (*P*=0.00783; Figure 6g). Cryptotanshinone alone did not affect IL10-mediated rescue of starvation-induced p-mTOR inhibition, whereas it inhibited IL10-mediated rescue of p-mTOR expression when combined with rapamycin in the presence of IL10. Rapamycin alone blocked IL10-mediated rescue of starvation-induced p-mTOR inhibition (Figure 6g). These results further indicate the involvement of both the AKT-mTOR and IL10R-STAT3 pathways in IL10-mediated inhibition of autophagy in starved HSFs.

### IL10 inhibits autophagy via the mTOR-p70S6K pathway

**Figure 7:**
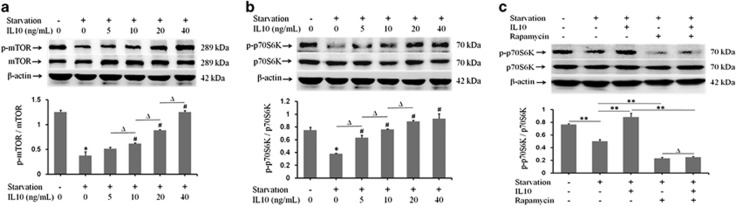
IL10-mediated inhibition of autophagy via the mTOR-p70S6K pathway. HSFs (70–80% confluent) were starved by culture in serum-depleted medium for 12–16 h. p-mTOR and p-p70S6K protein expression in response to IL10 treatment was then assessed in the presence or absence of rapamycin for 30 min. (**a**) p-mTOR and mTOR protein expression and changes in the p-mTOR/mTOR ratio in the presence of IL10 (data are expressed as the mean±S.E.M.; *n*=3, *P<0.05 *versus* the non-starvation group and ^#^*P*<0.05 *versus* the starvation group; Δ*P*<0.05). (**b**) p-p70S6K and p70S6K protein expression and changes in the p-p70S6K/p70S6K ratio in the presence of IL10 (*n*=3; *P<0.05 *versus* the non-starvation group and ^#^*P*<0.05 *versus* the starvation group; Δ*P*<0.05). (**c**) p-p70S6K and p70S6K protein expression and changes in the p-p70S6K/p70S6K ratio in the presence of IL10 and/or rapamycin (*n*=3; **P<0.001 *versus* the non-starvation control and **P<0.001 *versus* the starvation group; Δ*P*>0.05 *versus* the rapamycin-treatment group)

Phosphorylated p70S6K (p-p70S6K) is a signaling molecule that acts downstream of mTOR. As shown in Figures 7a and b, starvation inhibited the expression of both p-mTOR and p-p70S6K; however, IL10 rescued the expression of p-mTOR (*P*=0.16128, *P*=0.03496, *P*=0.00086, and *P*=0.00009; Figures 7a) and p-p70S6K (*P*=0.00023, *P*=0.00006, *P*=0.00000, and *P*=0.00016; Figure 7b) from starvation-induced inhibition in HSFs in a dose-dependent manner. Rapamycin strongly inhibited p-p70S6K expression (*P*=0.00007; Figure 7c), even in the presence of IL10 (*P*=0.00079; Figure 7c), but this effect was irreversible in the presence of IL10 (*P*=0.23409; Figure 7c). This result indicates that p-mTOR likely acts as a signaling molecule on which upstream signals converge before being transmitted downward to p-p70S6K.

## Discussion

HS formation usually results from disturbance of the tightly controlled tissue repair mechanism due to traumatic skin injury.^8, 12^ HS is not only aesthetically displeasing, but also obstructs normal muscle function, thereby contributing to psychological and physical suffering.^6, 7, 8, 9^

Autophagy is a lysosomal degradation pathway essential for cellular survival, differentiation, development, and homeostasis.^17, 40, 41, 42^ During disease pathogenesis, autophagy principally serves an adaptive role to protect organisms from pathogen infection and from aging, neurodegeneration, and cancer.^16, 18, 19, 20, 21, 22^ LC3 is an indicator of autophagy induction in mammals. LC3 typically localizes in the cytosol under normal conditions and translocates to autophagosomal membranes when autophagy is induced.^43, 44^ There are two forms of LC3: LC3-I and LC3-II. LC3-I is the non-lipidated form, with a molecular weight of 18 kDa, and LC3-II is the lipidated form, with a molecular weight of 16 kDa.^45, 46^ Conversion of LC3-I to LC3-II correlates with the formation of autophagosomes.^43, 44, 47^ Thus, changes in the LC3-II/LC3-I ratio are indicative of autophagic activity.

Currently, there is no effective therapy for HS, largely because the mechanisms underlying HS development are poorly understood.^10, 48^ IL10 was identified as a promising therapeutic agent that can reduce HS.^31, 32^ To clearly elucidate the mechanisms underlying the effects of IL10 on autophagy and HS formation, we examined autophagy in HSFs and NSFs in the presence or absence of IL10. The results showed that starvation can induce autophagic protein (Supplementary Figure 1) and mRNA (Supplementary Figure 2) expression in both HSFs and NSFs, and that this can be inhibited by IL10. As expected, the difference was more noticeable in HSFs than in NSFs (Supplementary Figures 1 and 2). The results also suggest (albeit indirectly) that IL10 has potential therapeutic benefit for the prevention and reduction of HS formation. Thus, we used HS tissue and serum-starved HSFs as our research platform.

IL10 is thought to function via the STAT3-mediated signaling pathway.^11, 35, 36^ Specifically, dimerized IL10 binds to the IL10R complex, which comprises two IL10R *α*-chains (IL10R*α*) and two accessory IL10R *β*-chains (IL10R*β*). IL10R*α* is unique to IL10R, whereas IL10R*β* is more diverse, being involved in signaling pathways related to several cytokines, including IL-22, IL-26, an x-interferon (*λ*-IFN), IL-28 A/B, and IL-29.^11, 36^ Therefore, preventing the inhibition of IL10R*α* functions would specifically disrupt IL10 functions that depend on IL10R*α*. STAT3 is a signaling molecule that interacts with multiple factors. STAT3 is phosphorylated by JAK1 and Tyk2, resulting in its dimerization and translocation to the nucleus to activate target gene expression.^11, 35, 36, 49^ To date, it is unclear whether the IL10R-STAT3 pathway is the primary mediator of IL10 function.^39, 50^

The AKT-mTOR pathway is responsible for cell survival, energy metabolism, and protein synthesis.^37, 38^ The results presented herein showed that IL10 increased STAT3 phosphorylation at Tyr705 and AKT phosphorylation at Ser473 (Figures 3a and b) in a dose-dependent manner. Treatment of HSFs with IL10RB inhibited p-STAT3 (Figure 4a) and p-AKT (Figure 4b) expression, whereas treatment of HSFs with the PI3K blocker, LY294002, downregulated both p-AKT (Figure 5a) and p-STAT3 (Figure 5b). These results imply that IL10 induces cross talk between the IL10R-STAT3 and AKT-mTOR pathways in starvation-treated HSFs.

To confirm whether IL10 inhibits autophagy via cross talk between the AKT-mTOR and IL10R-STAT3 pathways, HSFs were treated with various combinations of IL10, IL10RB, LY294002, cryptotanshinone, and rapamycin. IL10-mediated inhibition of autophagy was partly fortified by IL10RB, LY294002, cryptotanshinone, and rapamycin (Figures 4c,5c,4g, and 5g); however, IL10-mediated inhibition of autophagy was significantly increased by various combinations of these agents (Figures 6d and h). These data further corroborated the hypothesis that IL10 inhibits starvation-induced autophagy in HSFs by inducing p-AKT, p-STAT3, and p-mTOR expression via cross talk between the AKT-mTOR and IL10R-STAT3 pathways.

The mTOR kinase-dependent signaling pathway regulates autophagy.^51^ Activating the AKT-mTOR pathway inhibits autophagy, whereas the loss of signaling through this cascade removes the negative repression of mTOR.^52^ Therefore, there is a direct link between autophagy and the mTOR signaling pathway. Consistent with previous observations that p-mTOR activates the p70S6K complex leading to the inhibition of autophagy,^51, 52^ our data demonstrate that p-p70S6K was induced in starvation-treated HSFs exposed to IL10 (Figure 7b). Interestingly, p-p70S6K expression was abrogated by rapamycin in the presence of IL10 (Figure 7c). The LC3-II/LC3-I ratio increased during starvation, a phenomenon that was not reversed by rapamycin, even in the presence of IL10 (Figure 5g). These results confirmed that IL10 signaling activates mTOR and P70S6K (Figures 7a–c) to inhibit autophagy in starved HSFs.

**Figure 8:**
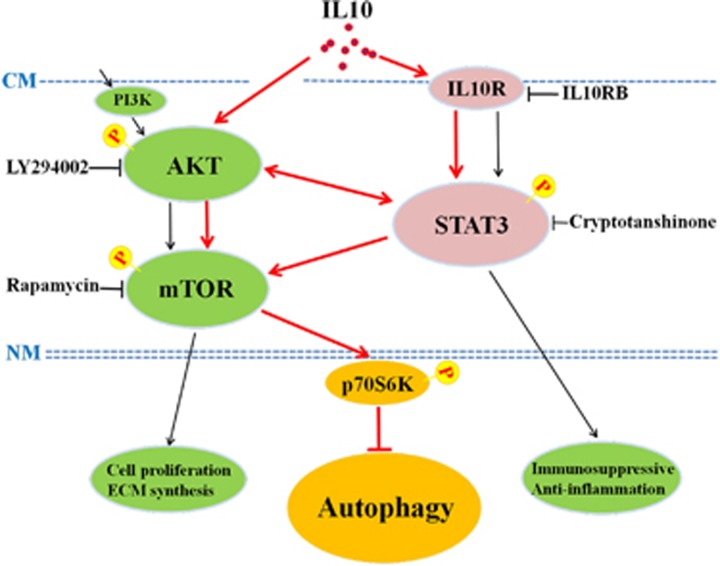
Schematic diagram showing the proposed mechanism underlying IL10-mediated inhibition of autophagy in starvation-treated HSFs. IL10 inhibits starvation-induced autophagy via IL10R-mediated activation of the IL10R-STAT3 pathway (IL10/IL10R-STAT3 pathway) or via direct activation of AKT-mTOR pathway (IL10/AKT-mTOR pathway). IL10 inhibits starvation-induced autophagy by inducing cross talk among STAT3, AKT, and mTOR, particularly STAT3 and mTOR and, finally, via activation of p70S6K ('→' activation, '⊥' inhibition)

In conclusion, we identified a novel mechanism by which IL10 inhibits autophagy in serum-starved HSFs. As illustrated schematically in Figure 8, IL10 inhibits starvation-induced autophagy through signaling via both the IL10/IL10R-STAT3 and IL10/AKT-mTOR pathways. Moreover, this phenomenon is likely mediated via the cross talk between STAT3, AKT, and mTOR, particularly between STAT3 and mTOR. This is further supported by our finding of significant increases in LC3 expression in starved HSFs in the presence of IL10 and cryptotanshinone (a STAT3 inhibitor) and rapamycin (an mTOR inhibitor) (Figures 6d and g). Taken together, these results strongly support the notion that IL10 inhibits starvation-induced autophagy in HSFs via cross talk between the IL10/IL10R-STAT3 and IL10/AKT-mTOR pathways (Figure 8). These findings shed light on the molecular mechanism underlying HS formation and highlight the therapeutic potential of IL10. Further studies based on cells harboring mutations in these signaling pathways may provide further insight into the role of IL10 in HS formation.

## Materials and Methods

### Collection and processing of HS tissue

HS and normal dermal skin (NS) tissues were collected from patients who had undergone surgical excision at Xijing Hospital (Xi'an, China). Written consent was obtained from all participants before surgery. All protocols used in the study were approved by the Ethics Committee of Xijing Hospital, affiliated to the Fourth Military Medical University of China. Each collected skin tissue sample was split into two portions: one portion was preserved in 10% buffered formalin solution for immunostaining and the remaining portion was to isolate fibroblasts for culture.

### Immunostaining

The skin tissues fixed in 10% buffered formalin were embedded in paraffin blocks and cut into 4*-μ*m-thick tissue sections. The processed tissue sections were then dewaxed and treated with 3% hydrogen peroxide for 15 min, followed by blocking with goat serum for 30 min, incubation at 4 °C overnight with a primary monoclonal antibody (mAb) against IL10R*α* (1:100 dilution; Santa Cruz Biotechnology, Dallas, TX, USA; 365374), and immunostained with a SP-9000 Histostain Kit (ZSGB, Beijing, China; SP-9000D), according to the manufacturer's instructions. Briefly, tissue sections were incubated with a biotinylated secondary antibody, treated with streptavidin-biotin-horseradish peroxidase for signal amplification, and then stained with diaminobenzidine. Finally, the tissue sections were counterstained with hematoxylin. Isotype-matched IgG was used as a negative control for each immunostaining procedure.

Immunofluorescence analysis was performed as previously reported.^9^ In brief, cells were grown on coverslips for 24–36 h until 70–80% confluent, fixed in 4% formaldehyde for 30 min, washed with phosphate-buffered saline (PBS), permeablized with 0.1% Triton-X100 for 10 min at room temperature, blocked with 1% bovine serum albumin, hybridized with a mouse mAb specific for IL10R*α* (1:500 dilution; Santa Cruz; 365374) at room temperature for 1 h, and then incubated with a Cy3-conjugated goat anti-mouse secondary antibody (1:100 dilution; Cwbio, Beijing, China; CW0159) at 37 °C for 1 h. Finally, the samples were stained with 4′,6′-diamidino-2-phenylindole (Sigma, St Louis, MO, USA; D9542).

### Transmission electron microscopy

Ultrathin sections of HSFs were processed in conventional methods. The samples were examined and imaged using a JEM-123 transmission electron microscope (JEOL, Tokyo, Japan) at 80 kV.

### Cell culture and treatment

Cell culture was performed as previously described.^5, 9^ Briefly, fibroblasts were extracted from minced HS tissues by incubation in a solution of collagenase type I (0.1 mg/ml; Sigma; C0130) at 37 °C for 2.5 h. Extracted HSFs were collected and cultured at 37 °C (in a 5% (v/v) CO_2_-humidified incubator) in Dulbecco's modified Eagle's medium (Gibco, Grand Island, NY, USA; 8113013) supplemented with 10% fetal calf serum (FCS; Gibco; 1087263), 100 U/ml penicillin, and 100 U/ml streptomycin (Hyclone, Logan, VT, USA; SV30010). All experiments were performed with cells at passage 3–5.

Biochemical analysis was conducted on HSFs at 70–80% confluence after incubation for 12–16 h in serum-free medium. Phosphorylation of STAT3, AKT, mTOR, and p70S6K was examined in HSFs treated with IL10 (10 ng/ml; PeproTech, Rocky Hill, NJ, USA; 0903B21-3), IL10RB (1:500 dilution; Santa Cruz; 365374), LY294002 (50 *μ*M; Beyotime, Haimen, Jiangsu, China; S1737), cryptotanshinone (4.6 *μ*M; Selleckchem, Houston, TX, USA; S2285), or rapamycin (1 *μ*g/*μ*l; Enzo, Farmingdale, NY, USA; BML-A275) for 30 min. Autophagy analysis (LC3 gene and protein expression) was conducted on HSFs treated for 6 h with each of the above reagents.

### qRT-PCR and PCR

qRT-PCR was performed as previously reported.^4, 9^ In brief, total RNAs were extracted from cultured cells using an RNA isolation kit (Takara, Dalian, Liaoning, China; 9109). The purity of the RNA was calculated as follows: A260/A280 (1.9–2.0). The primer sets used were as follows: LC3, forward 5′-CAACATGAGCGAGTTGGTCAAGA-3′ and reverse 5′-ACTCACCATGCTGTGCTGGTTC-3′ Beclin1, forward 5′-ATGCAGGTGAGCTTCGTGTG3′ and reverse 5′-CTGGGCTGTGGTAAGTAATGGA-3′ Atg5, forward 5′-GCTGCACTTTATTACCAAGCCTCTG-3′ and reverse 5′-AGCGTACTCAAATGGGTCAACATTC-3′ Atg12, forward 5′-AGTAGAGCGAACACGAACCATCC-3′ and reverse 5′-CCACGCCTGAGACTTGCAGTAA-3′ and GAPDH, forward 5′-GCACCGTCAAGCTGAGAAC-3′ and reverse 5′-TGGTGAAGACGCCAGTGGA-3′. The relative expression of the target gene transcripts was expressed as the mean abundance from three independent reactions. Expression was normalized against that of GAPDH.

To detect IL10R*α*, IL10R*α* was amplified by PCR using primers 5′-GCGAGATCTATGCTGCCGTGCCTCGTAGTGC-3′ (upstream) and 5′-CAGGGTACCTCACTCACTTGACTGCAGGCTAGAGAT-3′ (downstream) (*Bgl*II and *Kpn*I restriction sites underlined). The PCR products were then cloned into the *p*MD18T vector (Takara, 6011) and sequenced (Sangon, Shanghai, China).

### Western blot analysis

Cultured HSFs were harvested, washed in PBS, and resuspended in RIPA cell lysis solution (Beyotime; P0013C) supplemented with 200 *μ*g/ml phenylmethylsulfonyl fluoride (Boster, Wuhan, Hubei, China; AR1179), phosphatase inhibitor cocktail (Sigma; P0044), and protease inhibitor cocktail (Sigma; P8340). The protein concentration of the cell lysates was determined using the BCA assay (Pierce, Rockford, IL, USA; #23225).

Western blotting was performed as previously described.^9, 53^ Briefly, cell lysates containing equal amounts of protein were separated in 7% (for phosphorylated products) or 14% (for LC3) SDS-PAGE gels and transferred to polyvinylidene fluoride (Millipore, Temecula, CA, USA; ISEQ00010) membranes at 100 V for 25 min (for LC3) or 100 min (for mTOR, p70S6K, STAT3, and AKT). Membranes were then blocked with 5% non-fat milk in TBST (Tris-buffered saline/0.5% Tween-20) at room temperature for 3 h, followed by incubation at 4 °C overnight with rabbit mAbs specific for IL10R*α* (Epitomics, Burlingame, CA, USA; GTX102731), p-STAT3 (CST, #9145), STAT3 (CST, #12640), p-AKT (CST, #4060), AKT (CST, #4691), p-mTOR (CST, #5536), mTOR (CST, #2983), p-P70S6K (CST, #9234), p70S6K (CST, #9202), or LC3B (CST, Danvers, MA, USA; #3868). Finally, the membranes were washed and incubated with HRP-conjugated secondary antibodies (1:3000 dilution; Bioss, Beijing, China; bs-0295G-HRP). The immunoreactive protein bands were detected using ECL reagents (Millipore; WBKLS0100). The signal intensity of each protein was quantified by scanning the membrane with an image analyzer (Alpha Innotech, San Leandro, CA, USA). The membrane was then stripped of antibodies and re-probed with a rabbit mAb against *β*-actin (1:2000 dilution; CST; #4970) as an internal loading control.

### Statistical analysis

Quantitative data are expressed as the mean±standard error of the mean (S.E.M.). Student's *t-*test was used to compare data between two groups and analysis of variance was used for multiple-group comparisons. A *P*-value of <0.05 was considered statistically significant.

## Abbreviations

AKT: protein kinase B
BCA: bicinchoninic acid
DAB: diaminobenzidine
DAPI: 4′,6′-diamidino-2-phenylindole
ECL: enhanced chemiluminescence
ECM: extracellular matrix
FCS: fetal calf serum
GAPDH: glyceraldehyde-3-phosphate dehydrogenase
HRP: horseradish peroxidase
HS: hypertrophic scar
HSFs: hypertrophic scar derived fibroblasts
IL10: interleukin 10
IL10R: receptor of interleukin 10
IL10R*α*: receptor of interleukin 10 *α*-chain
IL10R*β*: receptor of interleukin 10 *β*-chain
IL10RB: function-blocking antibody against the receptor of interleukin 10 *α*-chain
IgG: immunoglobulin G
mAb: monoclonal antibody
LC3: microtubule-associated protein 1 light chain 3
mTOR: mechanistic target of rapamycin
NS: normal skin
NSFs: normal skin-derived fibroblasts
PBS: phosphate-buffered saline
PCR: polymerase chain reaction
PI3K: phosphoinositide 3-kinase
p70S6K: P70S6 kinase
qRT-PCR: quantitative real-time polymerase chain reaction
SDS-PAGE: sodium dodecyl sulfate-polyacrylamide gel electrophoresis
S.E.M.: standard error of the mean
STAT3: signal transducers and activators of transcription 3
TBST: tris-buffered saline/0.5% tween-20

## References

[bib1] Baehrecke EH. Autophagy: dual roles in life and death? Nat Rev Mol Cell Biol 2005; 6: 505–510. 15928714 10.1038/nrm1666

[bib2] Yang Z, Klionsky DJ. An overview of the molecular mechanism of autophagy. Curr Top Microbiol Immunol 2009; 335: 1–32. 19802558 10.1007/978-3-642-00302-8_1 PMC2832191

[bib3] Sidgwick GP, Bayat A. Extracellular matrix molecules implicated in hypertrophic and keloid scarring. J Eur Acad Dermatol Venereol 2012; 26: 141–152. 21838832 10.1111/j.1468-3083.2011.04200.x

[bib4] Zhang ZF, Zhang YG, Hu DH, Shi JH, Liu JQ, Zhao ZT et al. Smad interacting protein 1 as a regulator of skin fibrosis in pathological scars. Burns 2011; 37: 665–672. 21236582 10.1016/j.burns.2010.12.001

[bib5] Campaner AB, Ferreira LM, Gragnani A, Bruder JM, Cusick JL, Morgan JR. Upregulation of TGF-β1 expression may be necessary but is not sufficient for excessive scarring. J Invest Dermatol 2006; 126: 1168–1176. 16498396 10.1038/sj.jid.5700200

[bib6] Bayat A, McGrouther DA, Ferguson MW. Skin scarring. BMJ 2003; 326: 88–92. 12521975 10.1136/bmj.326.7380.88 PMC1125033

[bib7] Brown BC, McKenna SP, Siddhi K, McGrouther DA, Bayat A. The hidden cost of skin scars: quality of life after skin scarring. J Plast Reconst Aesthet Surg 2008; 61: 1049–1058. 10.1016/j.bjps.2008.03.020 18617450

[bib8] Aarabi S, Longaker MT, Gurtner GC. Hypertrophic scar formation following burns and trauma: New Approaches to Treatment. PloS Med 2007; 4: e234. 17803351 10.1371/journal.pmed.0040234 PMC1961631

[bib9] Shi JH, Guan H, Shi S, Cai WX, Bai XZ, Hu XL et al. Protection against TGF-β1-induced fibrosis effects of IL10 on dermal fibroblasts and its potential therapeutics for the reduction of skin scarring. Arch Dermatol Res 2013; 305: 341–352. 23321694 10.1007/s00403-013-1314-0

[bib10] Gauglitz GG, Korting HC, Pavicic T, Ruzicka T, Jeschke MG. Hypertrophic scarring and keloids: pathomechanisms and current and emerging treatment strategies. Mol Med 2011; 17: 113–125. 20927486 10.2119/molmed.2009.00153 PMC3022978

[bib11] Niessen FB, Spauwen PH, Schalkwijk J, Kon M. On the nature of hypertrophic scars and keloids: a review. Plast Reconstr Surg 1999; 104: 1435–1458. 10513931 10.1097/00006534-199910000-00031

[bib12] van der Veer WM, Bloemen MC, Ulrich MM, Molema G, van Zuijen PP, Middelkoop E et al. Potential cellular and molecular causes of hypertrophic scar. Burns 2009; 35: 15–29. 18952381 10.1016/j.burns.2008.06.020

[bib13] Wolfram D, Tzankov A, Pülzl P, Piza-Katzer H. Hypertrophic scars and keloids-a review of their pathophysiology, risk factors, and therapeutic management. Dermatol Surg 2009; 35: 171–181. 19215252 10.1111/j.1524-4725.2008.34406.x

[bib14] Tommasino C, Marconi M, Ciarlo L, Matarrese P, Malorni W. Autophagic flux and autophagosome morphogenesis require the participation of sphingolipids. Apoptosis 2015; 20: 645–657. 25697338 10.1007/s10495-015-1102-8

[bib15] Chang NC, Nguyen M, Bourdon J, Risse PA, Martin J, Danialou G et al. Bcl-2-associated autophagy regulator Naf-1 required for maintenance of skeletal muscle. Hum Mol Genet 2012; 21: 2277–2287. 22343142 10.1093/hmg/dds048

[bib16] Jin S, White E. Tumor suppression by autophagy through the management of metabolic stress. Autophagy 2008; 4: 563–566. 18326941 PMC2857579

[bib17] Levine B, Kroemer G. Autophagy in the pathogenesis of disease. Cell 2008; 132: 27–42. 18191218 10.1016/j.cell.2007.12.018 PMC2696814

[bib18] Orvadahl A, Levine B. Eating the enemy within: autophagy in infectious diseases. Cell Death Differ 2009; 16: 57–69. 18772897 10.1038/cdd.2008.130 PMC2736877

[bib19] Shintani T, Klionsky DJ. Autophagy in heath and disease: a double-edged sword. Science 2004; 306: 990–995. 15528435 10.1126/science.1099993 PMC1705980

[bib20] Todde V, Veenhuis M, van der Klei IJ. Autophagy: principles and significance in health and disease. Biochim Biophy Acta 2009; 1792: 3–13. 10.1016/j.bbadis.2008.10.016 19022377

[bib21] Winslow AR, Rubinsztein DC. Autophagy in neurodegeneration and development. Biochim Biophys Acta 2008; 1782: 723–729. 18644437 10.1016/j.bbadis.2008.06.010 PMC2597715

[bib22] Yen WL, Klionsky DJ. How to live long and prosper: autophagy, mitochondria, and aging. Physiology 2008; 23: 248–262. 18927201 10.1152/physiol.00013.2008

[bib23] Shi JH, Hu DH, Zhang ZF, Bai XZ, Wang HT, Zhu XX et al. Reduced expression of microtubule-associated protein 1 light chain 3 in hypertrophic scars. Arch Dematol Res 2012; 304: 209–215. 10.1007/s00403-012-1204-x 22237724

[bib24] Gutierrez MG, Master SS, Singh SB, Taylor GA, Colombo MI, Deretic V. Autophagy is a defense mechanism inhibiting BCG and Mycobacterium tuberculosis survival in infected macrophages. Cell 2004; 119: 505–517. 10.1016/j.cell.2004.11.038 15607973

[bib25] Pyo JO, Jang MH, Kwon YK, Lee HJ, Jun JI, Woo HN et al. Essential roles of Atg5 and FADD in autophagic cell death: dissection of autophagic cell death into vacuole formation and cell death. J Biol Chem 2005; 280: 20722–20729. 15778222 10.1074/jbc.M413934200

[bib26] Arico S, Petiot A, Bauvy C, Dubbelhuis PF, Meijer AJ, Codogno P et al. The tumor suppressor PTEN positively regulates macroautophagy by inhibiting the phosphatidylinositol 3-kinase/protein kinase B pathway. J Biol Chem 2001; 276: 35243–35246. 11477064 10.1074/jbc.C100319200

[bib27] Fiorentino DF, Bond MW, Mosmann TR. Two types of mouse T helper cell. IV. Th2 clones secrete a factor that inhibits cytokine production by Th1 clones. J Exp Med 1989; 170: 2081–2095. 2531194 10.1084/jem.170.6.2081 PMC2189521

[bib28] Moore KW, O'Garra A, de Waal Malefyt R, Vieira P, Mosmann TR. Interleukin-10. Annu Rev Immunol 1993; 11: 165–190. 8386517 10.1146/annurev.iy.11.040193.001121

[bib29] Singer AJ, Clark RA. Cutaneous wound healing. N Engl J Med 1999; 341: 738–746. 10471461 10.1056/NEJM199909023411006

[bib30] Peranteau WH, Zhang L, Muvarak N, Badillo AT, Radu A, Zoltick PW et al. IL10 overexpression decrease inflammatoly mediators and promotes regenerative healing in an adult model of scar formation. J Invest Dermatol 2008; 128: 1852–1860. 18200061 10.1038/sj.jid.5701232

[bib31] Occleston NL, O'Kane S, Goldspink N, Ferguson MW. New therapeutics for the prevention and reduction of scarring. Drug Discovery Today 2008; 13: 973–981. 18824245 10.1016/j.drudis.2008.08.009

[bib32] Kieran I, Knock A, Bush J, So K, Metcalfe A, Hobson R et al. Interleukin-10 reduces scar formation in both animal and human cutaneous wounds: results of two preclinical and phase II randomized control studies. Wound Repair Regen 2013; 21: 428–436. 23627460 10.1111/wrr.12043

[bib33] Hinz B. Formation and function of the myofibroblast during tissue repair. J Invest Demmatol 2007; 127: 526–537. 10.1038/sj.jid.5700613 17299435

[bib34] Hinz B. The myofibroblast: paradigm for a mechanically active cell. J Biomech 2010; 43: 146–155. 19800625 10.1016/j.jbiomech.2009.09.020

[bib35] Sabat R, Grütz G, Warszawska K, Kirsch S, Witte E, Wolk K et al. Biology of interleukin-10. Cytokine Growth Factor Rev 2010; 21: 331–344. 21115385 10.1016/j.cytogfr.2010.09.002

[bib36] Glocker EO, Kotlarz D, Klein C, Shah N, Grimbacher B. IL10 and IL10 receptor defects in humans. Ann N Y Acad Sci 2011; 1246: 102–107. 22236434 10.1111/j.1749-6632.2011.06339.x

[bib37] Franke TF, Kaplan DR, Cantley LC. pI3K: downstream AKTion block apoptosis. Cell 1997; 88: 435–437. 9038334 10.1016/s0092-8674(00)81883-8

[bib38] Franke TF. Intracellular signaling by AKT: bound to be specific. Sci Signal 2008; 1: pe29. 18560018 10.1126/scisignal.124pe29

[bib39] Park HJ, Lee SJ, Kim SH, Han J, Bae J, Kim SJ et al. IL10 inhibts the starvation induced autophagy in macrophages via class I phosphatidylinositol 3-kinase (PI3K) pathway. Mol Immunol 2011; 48: 720–727. 21095008 10.1016/j.molimm.2010.10.020

[bib40] Meijer AJ, Dubbelhuis PF. Amino acid signaling and the integration of metabolism. Biochem Biophys Res Commun 2004; 313: 397–403. 14684175 10.1016/j.bbrc.2003.07.012

[bib41] Riddle DL, Gorski SM. Shaping and stretching life by autophagy. Dev Cell 2003; 5: 364–365. 12967556 10.1016/s1534-5807(03)00269-7

[bib42] Yoshimori T. Autophagy: a regulated bulk degradation process inside cells. Biochem Biophys Res Commun 2004; 313: 453–458. 14684184 10.1016/j.bbrc.2003.07.023

[bib43] Asanuma K, Tanida I, Shirato I, Ueno T, Takahara H, Nishitani T et al. MAP-LC3 a promising autophagosomal marker, is processed during the differentiation and recovery of podocytes from PAN nephrosis. FASEB J 2003; 17: 1165–1167. 12709412 10.1096/fj.02-0580fje

[bib44] Tanida I, Minematsu-Ikequchi N, Ueno T, Kominami E. Lysosomal turnover, but not a cellular level, of endogenous LC3 is a marker for autophagy. Autophagy 2005; 1: 84–91. 16874052 10.4161/auto.1.2.1697

[bib45] Kabeya Y, Mizushima N, Ueno T, Yamamoto A, Kirisako T, Noda T et al. LC3, a mammalian homologue of yeast Apg8p, is localized in autophagosome membranes after processing. EMBO J 2000; 19: 5720–5728. 11060023 10.1093/emboj/19.21.5720 PMC305793

[bib46] Tanida I, Nishitani T, Nemoto T, Ueno T, Kominami E. Mammalian Apg12p, but not the Apg12p-Apg5p conjugate, facilitates LC3 processing. Biochem Biophys Res Commun 2002; 296: 1164–1170. 12207896 10.1016/s0006-291x(02)02057-0

[bib47] Karim MR, Kanazawa T, Daigaku Y, Fujimura S, Miotto G, Kadowaki M. Cytosolic LC3 ratio as a sensitive index of macroautophagy in isolated rat hepatocytes and H4-II-E cells. Autophagy 2007; 3: 553–560. 17617739 10.4161/auto.4615

[bib48] Saito M, Yamazaki M, Maeda T, Matsumura H, Setoguchi Y, Tsuboi R. Pirfenidone suppresses keloid fibroblast-embedded collagen gel contraction. Arch Dermatol Res 2012; 304: 217–222. 22033529 10.1007/s00403-011-1184-2

[bib49] Donnelly RP, Dickensheets H, Finbloom DS. The interleukin-10 signal transduction pathway and regulation of gene expression in mononuclear phagocytes. J Interferon Cytokine Res 1999; 19: 563–573. 10433356 10.1089/107999099313695

[bib50] Agbanoma G, Li C, Ennis D, Palfreeman AC, Williams LM, Brennan FM. Production of TNF-α in macrophages activated by T cells, compared with lipopolysaccharide, uses distinct IL10-dependent regulatory mechanism. J Immunol 2012; 188: 1307–1317. 22219323 10.4049/jimmunol.1100625

[bib51] Jung CH, Ro SH, Cao J, Otto NM, Kim DH. mTOR regulation of autophagy. FEBS Lett 2010; 584: 1287–1295. 20083114 10.1016/j.febslet.2010.01.017 PMC2846630

[bib52] Abraham RT, Wiederrecht GJ. Immunopharmacology of rapamycin. Ann Rev Immunol 1996; 14: 483–510. 8717522 10.1146/annurev.immunol.14.1.483

[bib53] Hu X, Wang H, Liu J, Fang X, Tao K, Wang Y et al. The role of ERK and JNK signaling in connective tissue growth factor induced extracellular matrix protein production and scar formation. Arch Dermatol Res 2013; 305: 433–445. 23494140 10.1007/s00403-013-1334-9

