# Supplementary material for: IL10 inhibits starvation-induced autophagy in hypertrophic scar fibroblasts via cross talk between the IL10-IL10R-STAT3 and IL10-AKT-mTOR pathways
Source: Cell Death Dis. 2016 Mar 10;7(3):e2133–. doi: 10.1038/cddis.2016.44 (PMC4823945; doi:10.1038/cddis.2016.44)
Supplement: Supplementary Information [file cddis201644x1.pdf]

**Supplementary Material**

**Supplementary Figure 1.** Comparison of the starvation-induced autophagy in HSFs and NSFs. HSFs and NSFs with 70-80% confluence were starved by culturing in serum-free medium for 12-16 h before treatment with IL10 at 20 ng/ml for 48 h. **(A)** Representative immunoblots of LC3, Beclin1 and Atg5 was analyzed by Western blot. **(B)** LC3-II/LC3-I ratio, **(C)** Beclin1 relative expression level, **(D)** Atg5 relative expression level were calculated based on signal intensity (Data are expressed as the represents mean ± SEM. N = 3, $^{**}p < 0.01$ *vs.* the non-starvation group or IL10-treated group, $^{*}p < 0.05$ *vs.* IL10-treated group).

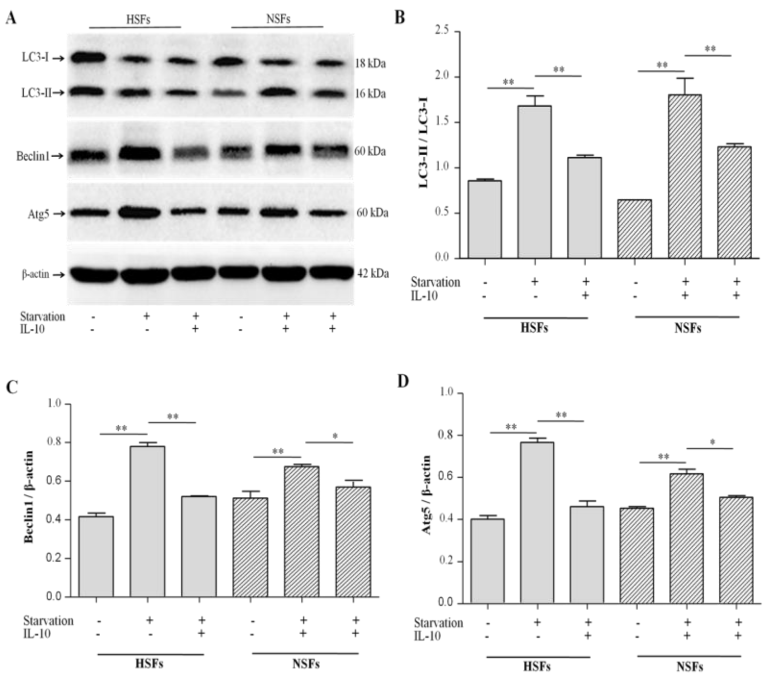


**Supplementary Figure 2.** Comparison of LC3B, Beclin1, Atg5 and Atg12 expressions on mRNA level between HSFs and NSFs. HSFs and NSFs were collected and RNA were extracted from each sample. The mRNA levels of fibrosis-related genes were quantified by qPCR. For all the experiments, mRNA values were normalized against corresponding GAPDH and presented as a ratio to control group (NS/NSFs group, arbitrarily set as 1). Data are expressed as the represents mean ± SEM. **(A)** LC3 mRNA, **(B)** Beclin1 mRNA, **(C)** Atg5 mRNA, and **(D)** Atg12 mRNA abundance was analyzed by qRT-PCR (Data are expressed as the mean ± SEM, n = 3, $^{**}p < 0.01$, $^{*}p < 0.05$, $^{\Delta}p > 0.05$ *vs.* the non-starvation group and IL10-treated group).

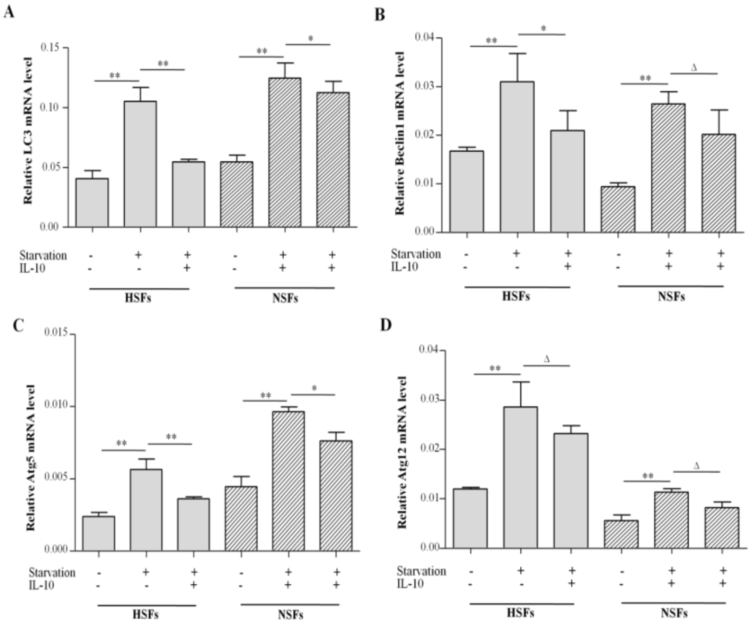

A
Relative LC3 mRNA level
0.15
0.10
0.05
0.00
**
**
**
*
Starvation
IL-10
HSFs
NSFs
B
Relative Beclin1 mRNA level
0.04
0.03
0.02
0.01
0.00
**
*
**
Δ
Starvation
IL-10
HSFs
NSFs
C
Relative Atg5 mRNA level
0.015
0.010
0.005
0.000
**
**
**
*
Starvation
IL-10
HSFs
NSFs
D
Relative Atg12 mRNA level
0.04
0.03
0.02
0.01
0.00
**
Δ
**
Δ
Starvation
IL-10
HSFs
NSFs
